# Post-stroke respiratory complications using machine learning with voice features from mobile devices

**DOI:** 10.1038/s41598-022-20348-8

**Published:** 2022-10-06

**Authors:** Hae-Yeon Park, DoGyeom Park, Hye Seon Kang, HyunBum Kim, Seungchul Lee, Sun Im

**Affiliations:** 1 Department of Rehabilitation Medicine, Seoul St. Mary’s Hospital, College of Medicine, The Catholic University of Korea, Seoul, Republic of Korea; 2 Graduate School of Artificial Intelligence, Pohang University of Science and Technology (POSTECH), Pohang, Republic of Korea; 3 Department of Pulmonary, Allergy and Critical Care Medicine, Bucheon St. Mary’s Hospital, College of Medicine, The Catholic University of Korea, Seoul, Republic of Korea; 4 Department of Internal Medicine, Bucheon St. Mary’s Hospital, College of Medicine, The Catholic University of Korea, Seoul, Republic of Korea; 5 Department of Otolaryngology-Head and Neck Surgery, Yeouido St. Mary’s Hospital, College of Medicine, The Catholic University of Korea, Seoul, Republic of Korea; 6 Department of Mechanical Engineering, Pohang University of Science and Technology (POSTECH), 223, 5th Engineering Building, 77 Cheongam-Ro, Nam-Gu, Pohang, 37673 Gyeongbuk Republic of Korea; 7 Department of Rehabilitation Medicine, Bucheon St. Mary’s Hospital, College of Medicine, Catholic University of Korea, 327 Sosa-ro, Seoul, Bucheon-si, 14647 Gyeonggi-do Republic of Korea

**Keywords:** Diagnostic markers, Biomedical engineering, Respiratory signs and symptoms, Gustatory system, Stroke

## Abstract

Abnormal voice may identify those at risk of post-stroke aspiration. This study was aimed to determine whether machine learning algorithms with voice recorded via a mobile device can accurately classify those with dysphagia at risk of tube feeding and post-stroke aspiration pneumonia and be used as digital biomarkers. Voice samples from patients referred for swallowing disturbance in a university-affiliated hospital were collected prospectively using a mobile device. Subjects that required tube feeding were further classified to high risk of respiratory complication, based on the voluntary cough strength and abnormal chest x-ray images. A total of 449 samples were obtained, with 234 requiring tube feeding and 113 showing high risk of respiratory complications. The eXtreme gradient boosting multimodal models that included abnormal acoustic features and clinical variables showed high sensitivity levels of 88.7% (95% CI 82.6–94.7) and 84.5% (95% CI 76.9–92.1) in the classification of those at risk of tube feeding and at high risk of respiratory complications; respectively. In both cases, voice features proved to be the strongest contributing factors in these models. Voice features may be considered as viable digital biomarkers in those at risk of respiratory complications related to post-stroke dysphagia.

## Introduction

Disturbed swallowing, or oropharyngeal dysphagia, commonly occurs after cerebrovascular disease, and may result in malnutrition, dehydration, and aspiration pneumonia^1^. These respiratory complications occur in approximately one-third of the post-stroke dysphagia population and are associated with high mortality and morbidity^2^. Early screening and prevention of these respiratory events may also affect prognosis; a recent study found that any previous episode of aspiration pneumonia resulted in poor stroke outcomes^3^.

Consequently, efforts have been made to develop a screening test that can safely and quickly predict aspiration pneumonia. Impaired gag reflex, dysphonia, weak cough, and choking after swallowing are known predictive factors^4^. Among these clinical signs, voice change is associated with aspiration and penetration^5^. A study has demonstrated that 90% of aspirators exhibit dysphonic vocal quality^6^, and specific voice patterns may indicate aspiration^7–9^. While speech-language pathologists and other experts can reliably detect pathological voice changes, non-experts can be inaccurate^10^ or miss them^5^. An acoustic analysis can increase the sensitivity of detecting voice changes objectively and help in clinical evaluation^11,12^.

Recently, machine learning (ML) and deep learning methods have been used to better predict voice disorders, achieving accuracy levels as high as 90% by using the acoustic parameters of jitter, shimmer, and noise-to-harmonic ratio (NHR)^13^. Another study using Gaussian mixture model system reported discriminating vocal fold disorders with 99% accuracy^14^. In addition, studies advocated using vocal biomarkers recorded via smartphones to classify patients with coronary artery disease and pulmonary hypertension^15–18^. By contrast, no study has used machine learning algorithms to identify those at risk of dysphagia and subsequent respiratory complications using vocal biomarkers. A previous study demonstrated that phonation among critically ill and intensive care unit patients helps screen aspiration, but objective acoustic features from the phonation data were not collected^19^. Also, despite the widespread use of mobile devices to integrate vocal biomarkers in making a patient’s diagnosis, no study has yet used these devices to analyze the voice features in these patients.

An automated system that identifies vocal biomarkers from those at risk of aspiration and thus require tube feeding in a non-invasive and easy manner using a mobile device has high potentials to be used at the bedside or in remote settings via telemedicine. Therefore, we aimed to determine if incorporating these digital voice signals, recorded via iPad tablets, into multimodal ML algorithms can accurately classify those at risk of aspiration. Because severe stroke can lead to aspiration pneumonia, we also sought to introduce the best model that classifies those at high risk of respiratory complications.

## Methods

### Study design and participants

This study included patients referred for swallowing disturbance for at least seven days at a university-affiliated hospital from September 2019 to June 2021. The inclusion criteria were participants with suspected swallowing disorder who were referred for swallowing assessment attributable to a brain lesion including stroke, and the ability to understand the instructions and participate in the phonation task. Participants who were unable to perform phonation or had not undergone the instrumental swallowing tests or other swallowing assessments were excluded from the study. Those with severe cognitive dysfunction that would not allow participation in the voice recording or spirometry assessment were excluded. Those with neurodegenerative disorders such as Parkinson’s disease, Alzheimer’s disease was also excluded. The Institutional Review Board (HC19EESE0060) of the Catholic University of Korea, Bucheon St. Mary’s hospital approved the use of pertinent clinical information relevant to swallowing, neurological deficit at the time of encounter, and medical record preceding the assessment to confirm for any respiratory events, including aspiration pneumonia for analysis. All the subjects’ identifying information was removed. Explanation of the study was provided to all participants verbally, with all pertinent information including the process of the voice recording. Voice recording was performed with patient’s consent during the routine swallowing assessment. After the data collection was completed, all personal information was de-identified. Any conversational component that would identify the participant were not recorded. Database was locked, and only researchers who were responsible for the software and data curation had authorization to access the data. Because this study presented no harm to subjects, took less than 2 min to complete and ensured participant privacy, the institutional review board approved the study. Informed consent was given by all participants for data collection.

### Datasets from voice recording

Voice recording was performed at enrollment with a blinded assessment, where the participants underwent clinical evaluation with chief complaints of dysphagia. Contrary to previous studies, no solid or liquid boluses were provided prior to voice recording. Voice was recorded with no oral bolus swallowing. Phonation were recorded using an iPad (Apple, Cupertino, CA, USA) through an embedded microphone. A voice recorder application by Apple was used, and the sound sampling frequency was 44,100 Hz. The digitized phonation signals were band-pass filtered between 20 and 8000 Hz to use data from the entire frequency band gathered by the iPad. In each case, the smart device was positioned 20 cm from the patient’s face^20^. Estimation accuracy is unaffected if the microphone is positioned within 30 cm from the participant’s mouth. Acoustic signals were obtained in a quiet room to eliminate ambient sounds^20^. No additional equipment was used. Under the examiner’s instructions, the patient was asked to phonate a single syllable for at least 5 s with comfortable pitch and loudness. To ensure uniformity in pitch variation a single instructor provided guidance so that the participants produced phonation under a comfortable pitch and loudness. An easy-to-follow single vowel phonation was chosen in this study in consideration that some participants manifested severe neurological deficits. The vowel /e/ vowel requires an unrounded lip position and a mid-tongue position and can be performed even in those with facial palsy or tongue deviations. A minimum of three attempts was recorded. The examiner was blinded to the patient’s neurological information and did not participate in the clinical evaluation of swallowing.

### Clinical parameters

Based on the findings from the instrumental swallowing test, enrolled participants were classified into two groups: (1) mild or minimal evidence of aspiration or dysphagia, who were deemed safe to undergo oral feeding, or (2) severe dysphagia with risk of aspiration that required tube feeding. The latter group was then further classified into two subgroups according to the risk of respiratory complications. This risk was stratified according to peak cough flow (PCF)^21^ evaluated using spirometry performed within the same day and abnormal chest x-ray images. Voluntary PCF was measured with forceful cough produced by the participants. Before the PCF measurement, the clinician provided verbal instructions to explain the method of cough production by command. The clinician then provided a live demonstration of coughing on the portable spirometer. Those with poor understanding could practice several times before a formal assessment. Voluntary PCF was measured on the peak flow meter (Micro-Plus Spirometer; Carefusion Corp.), in adherence to the guidelines recommended by the American Thoracic Society/European Respiratory Society^22^. The values were presented as the mean of the three highest values from the five attempts^23^. For voluntary PCF, cutoff values of less than 80 L/min were classified as high risk of respiratory complications^24^. Description on other clinical parameters is shown on Supplementary Methods S1.

### Preprocessing data

**Figure 1:**
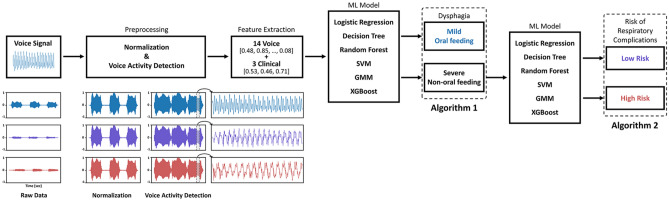
Algorithm development. Raw data from voice signals were preprocessed after normalization. Clinical data were concatenated to the *Praat* features in the machine learning models. A two step-process was then used to first classify those with oral feeding versus tube feeding (algorithm 1) and, among the latter, classify those at high risk of respiratory complications (algorithm 2). *ML* machine learning, *SVM* support vector machine, *GMM* Gaussian mixture model, *XGBoost* extreme gradient boosting.

The study used an Intel i9 X-Series processor and GeForce RTX 3090 (24 GB). A two-step wise model was developed using various ML algorithms that would classify (1) the presence of severe dysphagia requiring tube feeding and (2) high risk of respiratory complications with Fig. 1 delineating the preprocessing and model developmental process.

The preprocessing steps of data splitting, transformation and performance evaluation are presented in the Supplementary Methods S2.

### Feature extraction

The following features were extracted using the *Praat* software. Jitter and shimmer values can be measured using different parameters, namely local, absolute, Relative Average Perturbation (RAP), five point-Period Perturbation Quotient (PPQ5), and ddp for jitter; and local, localdbShimmer, Amplitude Perturbation Quotient (APQ)3, APQ5, APQ11, and Dda for shimmer values, and finally the cepstral peak prominence (CPP) values. Description of these features and feature selections are described in the Supplementary Methods S3.

### Multimodal model development

Age and severity of neurological deficit may affect voice features. To adjust these differences, clinical data were concatenated to the acoustic features in the ML algorithms to produce multimodal models. Among the various functional parameters, confounding variables were controlled according to the VIF, and those with high multicollinearity were excluded from the final models. To evaluate the effect of the clinical factors on the ML algorithms, we compared the addition of a clinical factor to the absence of one. The machine learning algorithms performed in this study are described in detail at the Supplementary Methods S4. All methods were carried out in accordance with relevant guidelines and regulation in model development and validation.

### Statistical analysis

Because participants were assessed at the time of enrollment, no missing data required the imputation method. The Shapiro–Wilk test for normality was used to evaluate the distribution of continuous variables. Between-group analyses were conducted using the Student’s t-test, Mann–Whitney test, or chi-square test. Continuous variables were expressed as mean and standard deviation, and categorical variables were expressed as numbers with percentages. All statistical analyses were performed using R Statistical Software (version 2.15.3; R Foundation for Statistical Computing, Vienna, Austria), which can be downloaded for free from the Internet (https://www.r-project.org/). For the ML algorithms, the performance of the prediction models was evaluated by computing the AUC, sensitivity, specificity, positive predictive value, and negative predictive value using the Scikit-Learn package in Python.

## Results

### Demographic features

A total of 449 participants were enrolled, and based on the instrumental swallowing tests, 215 participants (47.9%) were classified as having only mild dysphagia with mild aspiration that allowed full oral feeding, while 234 participants (52.1%) were classified as having severe dysphagia and aspiration and required tube feeding. As shown in Table 1, those with tube feeding exhibited severe neurological deficits and poor swallowing function, confirmed by the instrumental swallowing tests.

**Table 1:** Demographic features and acoustic parameters between mild versus severe dysphagia.

	Mild dysphagia (Oral feeding) (N = 215)	Tube feeding (Tube feeding) (N = 234)	*p *value
Age (years)	65.7 ± 13.2	72.2 ± 11.2	< 0.001*
Male	63.5 ± 13.9	71.4 ± 11.4	< 0.001*
Female	69.5 ± 11.1	73.4 ± 10.9	0.020*
Gender (Male)	135 (62.8%)	137 (58.5%)	0.411
Weight (kg)	61.3 ± 11.6	57.2 ± 10.8	< 0.001*
PAS	3.7 ± 1.9	7.2 ± 1.2	< 0.001*
Aspiration (Yes)	37 (17.2%)	222 (94.9%)	< 0.001*
FOIS	5.0 ± 1.0	1.7 ± 1.0	< 0.001*
MASA	182.6 ± 13.3	157.2 ± 17.3	< 0.001*
PCF (L/min)	231.7 ± 130.0	115.8 ± 87.8	< 0.001*
MMSE	22.9 ± 6.2	16.5 ± 8.7	< 0.001*
MBI	65.9 ± 29.4	29.8 ± 29.3	< 0.001*
NIHSS	5.3 ± 4.4	10.3 ± 5.1	< 0.001*
BBS	35.4 ± 20.1	15.2 ± 18.8	< 0.001*
F0 (Hz)	199.9 ± 58.8	206.0 ± 73.4	0.324
Male	183.7 ± 57.2	185.9 ± 67.7	
Female	227.1 ± 51.2	234.6 ± 71.9	
F0_SD (Hz)	5.0 ± 11.1	7.2 ± 12.5	0.051
HNR (dB)	16.45 ± 4.91	13.46 ± 5.36	< 0.001*
LocalJitter (%)	1.21 ± 0.98	2.03 ± 1.49	< 0.001*
LocalAbsoluteJitter (μs)	64.42 ± 55.12	112.64 ± 96.42	< 0.001*
RAP (%)	0.60 ± 0.52	1.05 ± 0.86	< 0.001*
PPQ5Jitter (%)	0.68 ± 0.60	1.18 ± 0.99	< 0.001*
DdpJitter (%)	1.80 ± 1.56	3.16 ± 2.59	< 0.001*
LocalShimmer (%)	6.71 ± 4.08	9.74 ± 5.47	< 0.001*
LocaldbShimmer	0.65 ± 0.34	0.91 ± 0.44	< 0.001*
APQ3Shimmer (%)	3.30 ± 2.02	5.04 ± 3.21	< 0.001*
APQ5Shimmer (%)	4.12 ± 3.05	6.12 ± 3.81	< 0.001*
APQ11Shimmer (%)	5.63 ± 4.83	8.05 ± 5.50	< 0.001*
DdaShimmer (%)	9.91 ± 6.07	15.14 ± 9.62	< 0.001*
CPP	23.36 ± 0.18	24.09 ± 0.15	< 0.001*

Values are presented in mean ± standard deviation (SD) or number (%).

*p < 0.05 is used for statistical significance.

*PAS* penetration-aspiration scale, *FOIS* functional oral intake scale, *MASA* mann assessment of swallowing ability, *PCF* peak cough flow, *MMSE* mini-mental state examination, *MBI* modified barthel index, *NIHSS* national institutes of health stroke scale, *BBS* berg balance scale, *F0* fundamental frequency, *HNR* harmonic to noise ratio, *RAP* relative average perturbation, *PPQ* period perturbation quotient, *APQ* amplitude perturbation quotient, *CPP* cepstral peak prominence.

**Figure 2:**
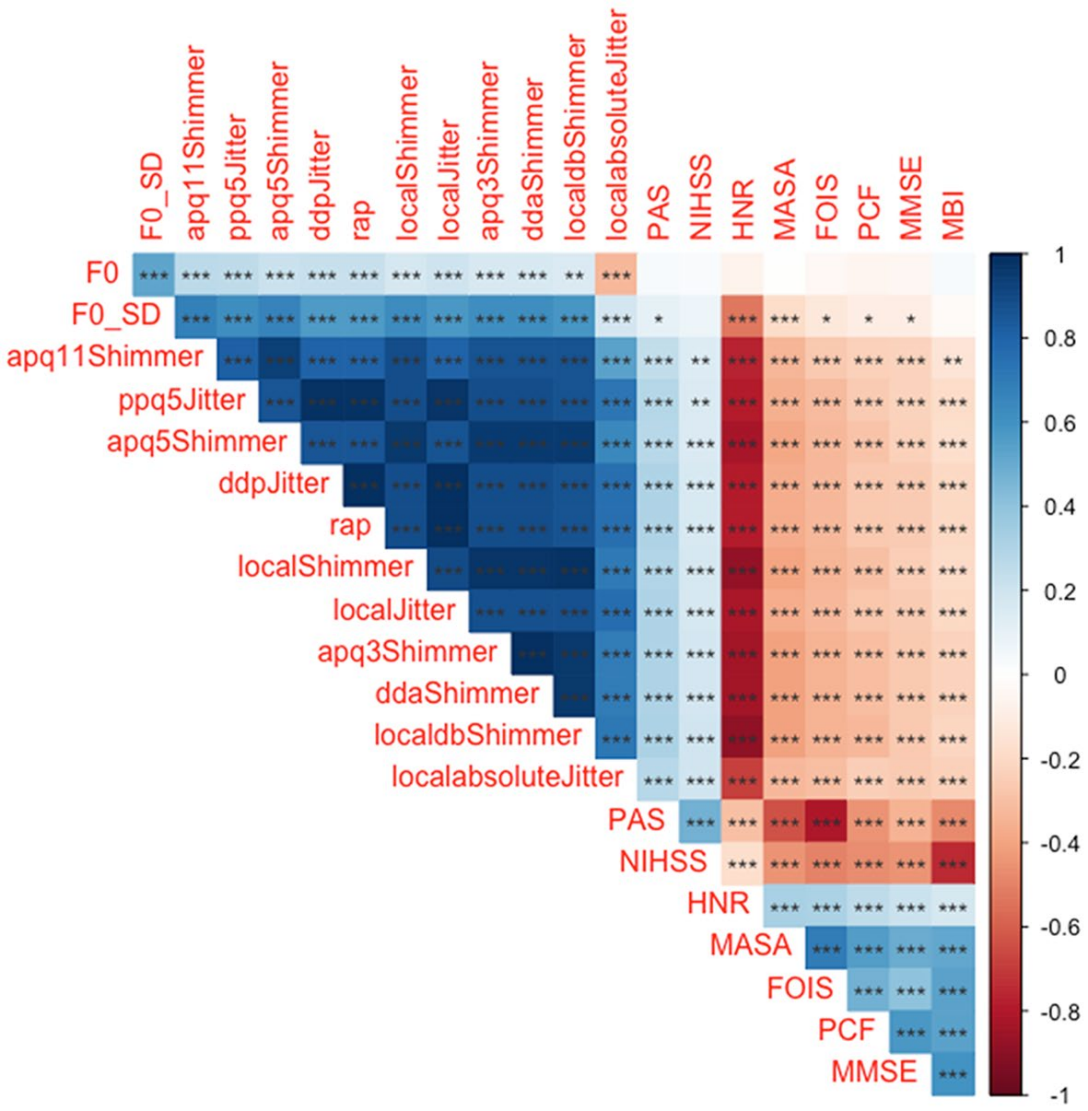
Correlation analysis between the Praat features and the clinical parameters. The correlation graph shows that nearly all the voice features showed significant association with the clinical parameters, especially with those related to swallowing, and peak cough flow values. An exception was observed with the fundamental frequencies, which failed to show any association with the clinical parameters. *p < 0.05; **p < 0.01; ***p < 0.001. *HNR* harmonic to noise ratio, *F0* fundamental frequency, *MBI* modified barthel index, *NIHSS* national institutes of health stroke scale. *F0* fundamental frequency, *SD* standard deviation, *APQ* amplitude perturbation quotient, *PPQ* period perturbation quotient, *RAP* relative average perturbation, *PAS* penetration-aspiration scale, *NIHSS* national institutes of health stroke scale, *HNR* harmonic to noise ratio, *MASA* mann assessment of swallowing ability, *FOIS* functional oral intake scale, *PCF* peak cough flow, *MMSE* mini-mental state examination, *MBI* modified barthel index.

Among those with tube feeding, 113 participants (48.3%) in the high risk (higher risk for respiratory complications) showed abnormal chest x-ray findings with significantly low voluntary cough strength (52.4 ± 20.0 L/min) compared to those with low risk (lower risk for respiratory complications) (175.1 ± 85.2 L/min) (Table 2). Among these patients, 93% (n = 105) had been linked to a respiratory disorder, such as confirmed aspiration pneumonia (n = 98) or pleural effusion or bronchitis (n = 7) after dysphagia onset. The SNR of the voice files were in the range 55–70 dB, which indicate that the background noise was minimal. The high-risk group showed higher values of standard deviation of the fundamental frequency, frequency and amplitude perturbation, and noise parameters than the low-risk group. Figure 1 shows how clinical data were concatenated to the acoustic features in the ML algorithms to produce multimodal models. Figure 2 shows the relationship between the voice features and clinical parameters.

**Table 2:** Demographic features and acoustic parameters according to respiratory complication risk within those with tube feedings.

	Low risk (N = 121)	High risk (N = 113)	*p* value
Age (years)	70.2 ± 12.1	74.4 ± 9.8	0.004*
Male	70.1 ± 12.3	73.7 ± 9.5	0.078
Female	70.4 ± 11.7	75.0 ± 10.1	0.044*
Gender (Male)	86 (71.1%)	51 (45.1%)	1.000
Weight (kg)	59.2 ± 9.9	55.0 ± 11.3	0.003*
PAS	7.1 ± 1.1	7.3 ± 1.2	0.131
FOIS	1.9 ± 1.0	1.6 ± 0.9	0.061
MASA	165.1 ± 13.4	148.6 ± 16.9	< 0.001*
PCF (L/min)	175.1 ± 85.2	52.4 ± 20.0	< 0.001*
MMSE	20.7 ± 7.5	11.9 ± 7.5	< 0.001*
MBI	41.6 ± 31.5	17.1 ± 20.3	< 0.001*
NIHSS	9.1 ± 5.3	11.6 ± 4.6	< 0.001*
BBS	21.9 ± 20.9	7.9 ± 12.8	< 0.001*
F0 (Hz)	199.0 ± 68.7	213.5 ± 77.7	0.13
Male	181.92 ± 60.32	192.48 ± 78.89	
Female	241.04 ± 70.78	230.88 ± 72.83	
F0_SD (Hz)	5.00 ± 9.27	9.48 ± 14.89	0.007*
HNR (dB)	14.29 ± 4.83	12.57 ± 5.76	0.013*
LocalJitter (%)	1.63 ± 1.08	2.45 ± 1.74	< 0.001*
LocalAbsoluteJitter (μs)	93.15 ± 74.20	133.51 ± 112.21	0.001*
RAP (%)	0.83 ± 0.62	1.29 ± 1.02	< 0.001*
PPQ5Jitter (%)	0.93 ± 0.71	1.44 ± 1.17	< 0.001*
DdpJitter (%)	2.49 ± 1.85	3.88 ± 3.05	< 0.001*
LocalShimmer (%)	8.38 ± 4.31	11.19 ± 6.19	< 0.001*
LocaldbShimmer	0.80 ± 0.36	1.03 ± 0.49	< 0.001*
APQ3Shimmer (%)	4.24 ± 2.37	5.91 ± 3.73	< 0.001*
APQ5Shimmer (%)	5.18 ± 2.80	7.13 ± 4.45	< 0.001*
APQ11Shimmer (%)	6.64 ± 3.25	9.55 ± 6.86	< 0.001*
DdaShimmer (%)	12.72 ± 7.10	17.73 ± 11.20	< 0.001*
CPP	23.81 ± 0.30	24.52 ± 0.37	< 0.001*

Values are presented in mean ± standard deviation (SD) or number (%).

*p < 0.05 is used for statistical significance.

*PAS* penetration-aspiration scale, *FOIS* functional oral intake scale, *MASA* mann assessment of swallowing ability, *PCF* peak cough flow, *MMSE* mini-mental state examination, *MBI* modified barthel index, *NIHSS* national institutes of health stroke scale, *BBS* berg balance scale, *F0* fundamental frequency, *HNR* harmonic to noise ratio, *RAP* relative average perturbation, *PPQ* period perturbation quotient, *APQ* amplitude perturbation quotient, *CPP* cepstral peak prominence.

### Severity of dysphagia, tube feeding risk classification (algorithm 1)

**Figure 3:**
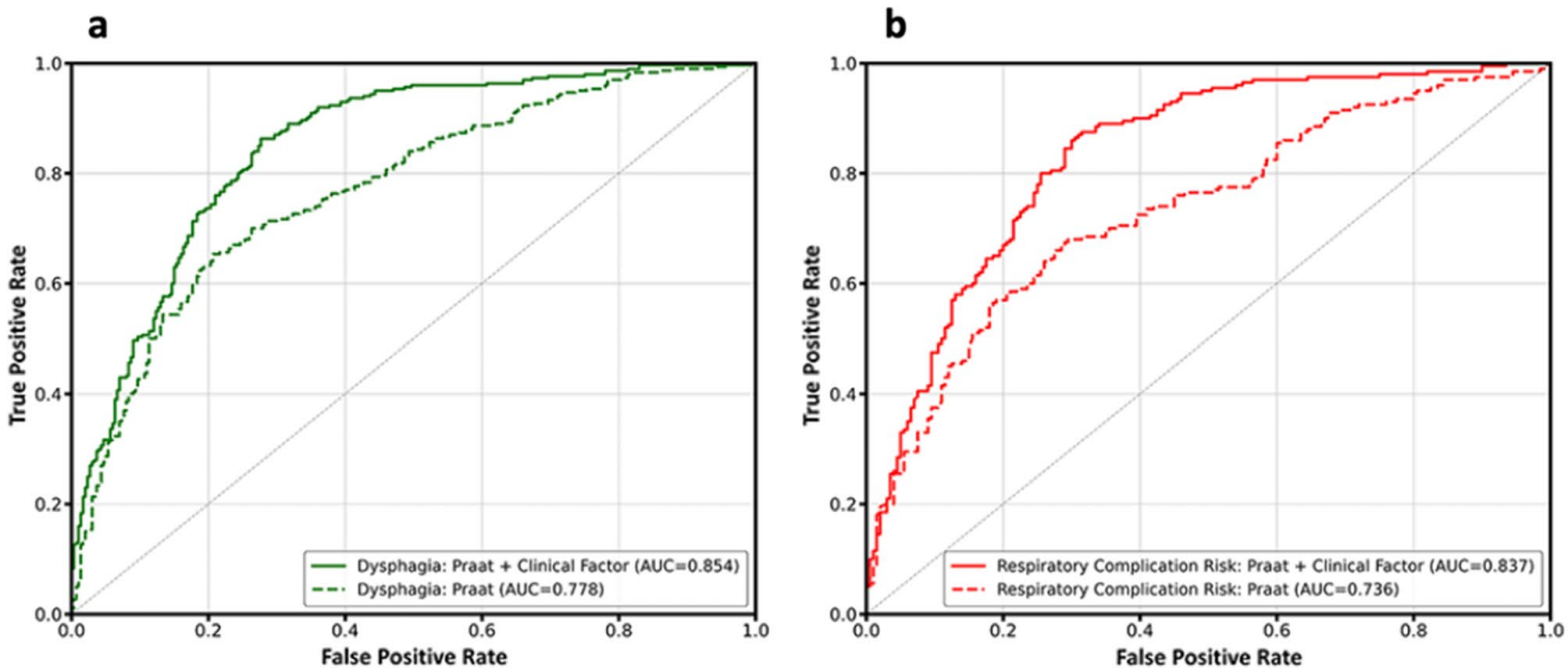
AUC-ROC curve of the XGBoost model for classifying tube feeding and risk of respiratory complications. AUC-ROC curves show that multimodal models that combine phonation and clinical data demonstrate high levels of AUC in classifying (**a**) risk of tube feeding and (**b**) respiratory complications. *AUC* area under curve, *ROC* receiver operating characteristic.

Table 3 shows that among the various ML models, the XGBoost model showed the highest sensitivity (72.7%; 95% CI 64.3–81.0%) and AUC (0.78; 95% CI 0.73–0.82) levels with the selected *Praat* features. After testing for multicollinearity, age, weight, and NIHSS score were selected to evaluate the performance of the models with the voice parameters. The multimodal models showed improved diagnostic properties, with the XGBoost model again showing the highest sensitivity (88.7%; 95% CI 82.6–94.7%) and AUC (0.85; 95% CI 0.82–0.89) values (Fig. 3a).

**Table 3:** Evaluation metric table of samples for voice signals in classifying tube feeding.

	Accuracy (%)	Sensitivity (%)	Specificity (%)	NPV (%)	PPV (%)	F1	AUC
**Voice only**							
LR	68.2 (64.3–72.1)	65.7 (58.2–73.1)	70.7 (65.4–75.9)	67.8 (63.1–72.6)	69.3 (65.0–73.7)	0.67 (0.62–0.72)	0.69 (0.64–0.74)
DT	69.0 (64.5–73.5)	62.0 (56.5–67.5)	76.0 (67.2–84.8)	66.6 (63.2–70.0)	73.3 (66.1–80.6)	0.67 (0.62–0.71)	0.70 (0.65–0.75)
RF	73.7 (70.2–77.1)	70.7 (66.1–75.3)	76.7 (70.5–82.8)	72.5 (69.2–75.7)	75.7 (70.8–80.6)	0.73 (0.69–0.76)	0.78 (0.73–0.82)
SVM	69.7 (65.9–73.5)	71.0 (66.9–75.1)	68.3 (62.8–73.9)	70.2 (66.4–74.0)	69.4 (65.2–73.5)	0.70 (0.67–0.74)	0.68 (0.63–0.73)
GMM	66.2 (61.3–71.0)	64.7 (51.3–78.1)	67.7 (60.1–75.2)	67.5 (61.2–73.7)	66.3 (61.1–71.5)	0.64 (0.55–0.74)	0.64 (0.55–0.72)
XGBoost	**74.8 (71.0–78.7)**	**72.7 (64.3–81.0)**	**77.0 (68.9–85.1)**	**74.8 (69.5–80.2)**	**76.8 (71.2–82.4)**	**0.74 (0.69–0.79)**	**0.78 (0.73–0.82)**
**Voice + clinical**							
LR	77.2 (74.4–80.0)	76.7 (67.1–86.2)	**77.7 (70.1–85.2)**	78.3 (72.5–84.2)	78.7 (73.5–83.8)	0.77 (0.73–0.81)	0.82 (0.79–0.85)
DT	74.5 (70.6–78.4)	80.0 (72.9–87.1)	69.0 (64.8–73.2)	78.3 (72.6–84.0)	72.1 (68.8–75.3)	0.76 (0.71–0.80)	0.75 (0.71–0.80)
RF	79.7 (75.9–83.4)	85.0 (78.6–91.4)	74.3 (67.0–81.7)	83.9 (78.9–89.0)	77.5 (72.3–82.7)	0.81 (0.77–0.84)	0.84 (0.80–0.88)
SVM	77.0 (73.8–80.2)	84.3 (78.7–90.0)	69.7 (64.1–75.2)	82.3 (77.4–87.1)	73.8 (70.4–77.2)	0.79 (0.75–0.82)	0.81 (0.77–0.84)
GMM	73.2 (68.9–77.5)	76.3 (69.5–83.1)	70.0 (63.6–76.4)	75.4 (69.4–81.3)	72.1 (67.6–76.6)	0.74 (0.70–0.78)	0.75 (0.71–0.79)
XGBoost	**82.5 (78.0–87.0)**	**88.7 (82.6–94.7)**	76.3 (71.4–81.3)	**87.6 (81.6–93.5)**	**79.0 (74.9–83.1)**	**0.83 (0.79–0.88)**	**0.85 (0.82–0.89)**

Values are presented in mean (95% confidence interval). Values with bold-text represent the highest values among the models.

*NPV* negative predictive value, PPV positive predictive value, *AUC* area under curve, *LR* logistic regression, *DT* decision tree, *RF* random forest, *SVM* support vector machine, *GMM* Gaussian mixture model, *XGBoost* extreme gradient boosting.

### Respiratory risk classification (algorithm 2)

Table 4 shows the performance of the ML algorithms for the risk classification of respiratory complications with *Praat* features. The XGBoost model's algorithms showed the highest sensitivity (76.5%; 95% CI 68.2%-84.8%) and AUC (0.74; 95% CI 0.66–0.81) levels. MBI was selected as a clinical feature to evaluate the risk of aspiration pneumonia, while other factors, including MMSE, were not used in this algorithm because of multicollinearity with other variances. With the inclusion of MBI, age and weight in the models, the sensitivity of the XGBoost model increased to 84.5% (95% CI 76.9–92.1%), and the AUC increased to 0.84 (95% CI 0.81–0.87) (Fig. 3b).

**Table 4:** Evaluation metric table of samples for voice signals in classifying risk of respiratory complications.

	Accuracy (%)	Sensitivity (%)	Specificity (%)	NPV (%)	PPV (%)	F1	AUC
**Voice only**							
LR	66.2 (61.5–71.0)	55.0 (49.5–60.5)	77.5 (70.5–84.5)	63.3 (59.4–67.2)	72.2 (65.1–79.3)	0.62 (0.56–0.67)	0.64 (0.59–0.70)
DT	70.0 (64.3–75.7)	72.5 (65.3–79.7)	67.5 (57.5–77.5)	71.5 (64.6–78.4)	70.2 (62.5–77.9)	0.71 (0.66–0.76)	0.71 (0.64–0.77)
RF	70.5 (66.5–74.5)	71.5 (60.4–82.6)	69.5 (57.9–81.1)	72.7 (66.5–78.8)	72.0 (65.1–79.0)	0.70 (0.65–0.75)	0.73 (0.67–0.78)
SVM	67.0 (63.4–70.6)	56.0 (47.9–64.1)	**78.0 (72.9–83.1)**	64.5 (60.3–68.7)	72.2 (66.8–77.6)	0.63 (0.57–0.68)	0.65 (0.61–0.69)
GMM	62.2 (57.8–66.7)	69.0 (62.7–75.3)	55.5 (45.9–65.1)	64.3 (59.7–68.9)	61.5 (56.5–66.4)	0.65 (0.61–0.68)	0.61 (0.55–0.66)
XGBoost	**74.0 (68.5–79.5)**	**76.5 (68.2–84.8)**	71.5 (59.3–83.7)	**76.4 (71.3–81.4)**	**75.9 (66.3–85.5)**	**0.75 (0.71–0.79)**	**0.74 (0.66–0.81)**
**Voice + clinical**							
LR	75.8 (71.3–80.2)	78.0 (70.4–85.6)	73.5 (64.1–82.9)	78.2 (71.1–85.3)	75.9 (68.9–82.8)	0.76 (0.72–0.80)	0.79 (0.74–0.84)
DT	73.8 (68.7–78.8)	74.5 (66.7–82.3)	73.0 (66.6–79.4)	74.8 (68.3–81.4)	73.7 (68.2–79.1)	0.74 (0.68–0.79)	0.73 (0.68–0.79)
RF	76.5 (73.1–79.9)	82.0 (77.6–86.4)	71.0 (64.1–77.9)	80.3 (75.7–84.9)	74.4 (69.8–79.1)	0.78 (0.75–0.81)	0.81 (0.77–0.86)
SVM	74.5 (70.4–78.6)	80.5 (70.0–91.0)	68.5 (59.6–77.4)	80.1 (72.0–88.2)	72.7 (68.2–77.2)	0.76 (0.71–0.80)	0.76 (0.72–0.80)
GMM	74.2 (69.7–78.8)	81.5 (75.1–87.9)	67.0 (57.6–76.4)	79.6 (73.3–85.9)	72.8 (65.5–80.2)	0.76 (0.72–0.80)	0.76 (0.71–0.81)
XGBoost	**80.8 (78.7–82.8)**	**84.5 (76.9–92.1)**	**77.0 (69.4–84.6)**	**84.6 (79.3–89.8)**	**79.8 (74.4–85.1)**	**0.81 (0.79–0.84)**	**0.84 (0.81–0.87)**

Values are presented in mean (95% confidence interval). Values with bold-text represent the highest values among the models.

*NPV* negative predictive value, PPV positive predictive value, *AUC* area under curve, *LR* logistic regression, *DT* decision tree, *RF* random forest, *SVM* support vector machine, *GMM* Gaussian mixture model, *XGBoost* extreme gradient boosting.

### Feature contribution

**Figure 4:**
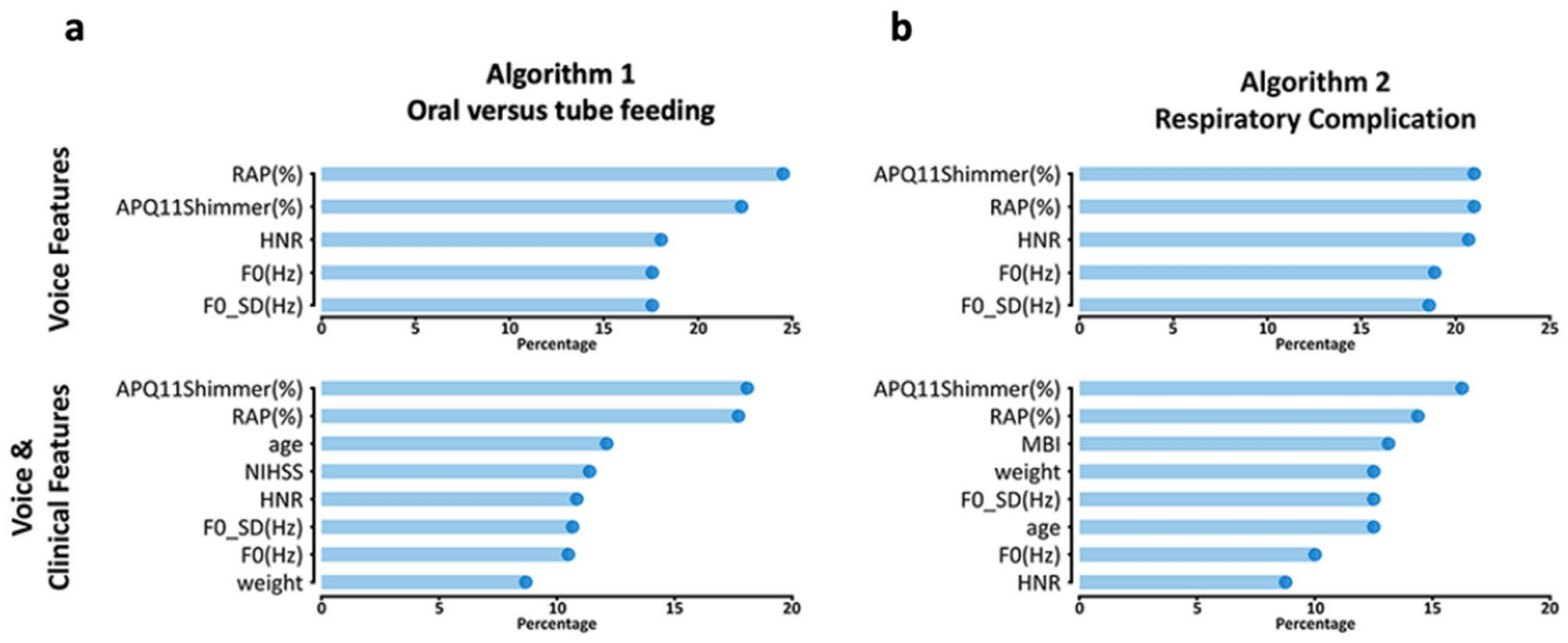
Feature importance analysis. Feature importance analysis from the XGBoost with plots demonstrating that APQ11Shimmer and RAP values are the major features even after including the clinical variables in (**a**) classifying those with tube feeding and (**b**) at risk of respiratory complications. *XGBoos*t extreme gradient boosting, *RAP* relative average perturbation, *APQ* amplitude perturbation quotient, *HNR* harmonic to noise ratio, *F0* fundamental frequency, *MBI* modified barthel index, *NIHSS* national institutes of health stroke scale.

Feature scoring is frequently used to interpret ML algorithms. The XGBoost algorithm counts out the importance by gain, frequency, and cover. Gain is the measured value of the contribution to each tree of an ensemble model. Cover is the relatively measured value of the observed value through the leaf node of each tree in the model. Frequency is the measured value as to how frequently each independent variable is used decisively in the model. We choose gain to calculate the feature importance score. Figure 4 shows how *Praat* and clinical features contributed to the classification in the XGBoost model. Among these, the RAP and APQ11Shimmer were major features, even after the inclusion of other clinical features to the model.

## Discussion

This study demonstrated that the acoustic parameters recorded via a mobile device may help distinguish post-stroke patients at high risk of respiratory complications. Among various ML models, the XGBoost multimodal model that included acoustic parameters, age, weight, and NIHSS score, showed an AUC of 0.85 and high sensitivity levels of 88.7% in the classification of those with tube feeding and high risk of aspiration. A second model showed an AUC of 0.84 and high sensitivity levels of 84.5% in the classification of those at risk of respiratory complications. Among these parameters, APQ11shimmer and RAP proved to be the strongest contributing factors. Our results are consistent with recent work advocating for the use of vocal biomarkers for various medical^15,16^ and neurological disorders. These algorithms could facilitate the early identification of those at risk of aspiration and help prevent respiratory complications in an automated and objective manner.

Comparative analysis of different data input types shows that a multimodal combination approach with clinical factors can improve model performance. Previous studies have advocated the combination of clinical and demographic data with voice signals to distinguish different voice pathologies^25^. Such multimodal learning models have proven successful in the classifying pathological voice disorders such as neoplasms, phonotrauma, and vocal palsy^26^. Considering that post-stroke pneumonia is multifactorial^27^, it may be too far-fetched to conclude that simple vowel phonation could classify aspiration risk, necessitating the development of multimodal algorithms. Following these previous studies, the multimodal models in this study showed the highest sensitivity level up to 88.7%. These were promising results considering that subjective methods in swallowing investigation can only detect 40% of aspiration occurrences^28^. The clinical factors used in this study were already known to predict the risk of aspiration pneumonia, with NIHSS known to be a useful parameter for predicting dysphagia in stroke patients^29,30^ and sub-score of MBI or levels of disability to be associated with aspiration pneumonia^27,31^.

One of the key characteristics of this study was to classify the risk of respiratory complications based on the spirometry findings. In contrast, previous studies have attempted to use acoustic features to classify the presence/absence of aspiration per se from instrumental assessments, which have shown inconsistent diagnostic properties^11,12^. As in phonation, complete glottic closure is an essential mechanism for generating a strong cough^32^. Smith-Hammond and Goldstein reported that objective measurements of voluntary cough could be used to help identify at-risk of aspiration in stroke patients^8^. Not all patients with abnormal swallow aspirate, with only one-third showing aspiration on VFSS^2,33,34^. Similarly, not all who aspirate develop aspiration pneumonia; only one-third do. Therefore, in this study, the risk of respiratory complication was not classified based on presence of aspiration but the strength of the voluntary cough and abnormal chest images. 93% of those in the high-risk group had confirmed aspiration pneumonia proving the validity of these measures to assess those at risk of respiratory complications.

Another important characteristic is that we incorporated acoustic features into the ML algorithms. Among these models, XGBoost showed the best accuracy and had higher learning potential than the other models, which was expected because of the structure that this technique is a nonlinear model with larger dimensions and has shown excellent performance in other clinical studies^35,36^. From these XGBoost algorithms, the APQ11shimmer and RAP were shown to be the two major *Praat* features even after controlling other clinical factors that may increase respiratory complications, indicating that these two features may be used as potential biomarkers to distinguish those at high-risk respiratory complications.

Past studies on which acoustic parameters can best predict aspiration^11^ have shown conflicting results^12^.

Our results showed consistent findings that the RAP and APQ11shimmer to be the most vital contributing factors in classifying those with tube feeding and those at high risk of respiratory complications, though some differences were observed in the rank of these features in each model. For example, the RAP was the most crucial contributor to classifying those with severe dysphagia that would require tube feeding, who also showed a more severe grade of an aspiration than those with mild dysphagia. RAP is one of the best jitter parameters that reflects the perceptual pitch and increases when the glottis is in contact with material or secretion^5^ and is therefore very likely to be the most significant contributor in classifying those at high risk of aspiration. Our results correspond to previous studies that have shown the RAP to show significant changes after aspiration^12^ with high sensitivity levels^11^.

By contrast, the APQ11 shimmer was the most critical contributor in classifying those at risk of respiratory complications. Our findings agree with the fact that among the amplitude perturbation parameters, APQ11shimmer is known to reflect better the decreased glottic control than the APQ3 or APQ5. Proper glottic closure is related to the cough force, and its impairment can lead to improper secretion clearance and aspiration pneumonia^32^. Our results support the role of APQ11shimmer as a marker that reflects glottic dysfunction that plays a most significant role in the model to classify those at high risk of respiratory complications. This strong association of APQ11shimmer with glottic dysfunction was further proven in a previous machine learning study classifying glottic cancer^37^. A final point of interest was while CPP has been pointed to provide a solid basis to quantitatively approach dysphonia severity, this was not observed in this study. The CPP showed high VIF values and therefore were not eligible to be included in the ML algorithms^38^.

In this study, voice recordings were easily attained with no adverse effects or technical difficulties. Recording with mobile devices eliminates the potential risk of bolus aspiration and does not require the use of special equipment, such as a sensor or accelerometer^39,40^. The methods proposed in this study differed from past studies in that the voice recording was performed without the introduction of additional bolus material and posed no additional threat of aspiration and thus can be safely performed even on those at risk of aspiration pneumonia. Also, the main objective of our algorithms differed from past studies; we attempted to classify those with severe dysphagia or risk of respiratory complications, while past studies attempted only to detect the presence of aspiration per se via voice changes after a bolus swallow. Additionally, voice recordings can easily be performed on severely ill patients to obtain objective voice biomarkers. In a broader context, this novel method to assess patients admitted to the intensive care unit or in the emergency setting, which require an easy but noninvasive bedside evaluation to screen for the risk of dysphagia and aspiration pneumonia^19^ before referral for instrumental tests.

Our study has some limitations. First, only those with brain lesions were included in the study; and those with any degree of dysphagia referred for assessment were used for model development with no comparison to a healthy normal population. However, our ongoing studies on deep learning models include acoustic analysis data from a healthy population and attempt to distinguish dysphagic voices from normal ones. Second, while those undergoing FEES underwent phonation recording simultaneously, there was a lapse in those who underwent VFSS with a median interval of four days. Future studies that assess voice change 24 h after stroke onset upon hospital arrival would better reflect early voice change in predicting dysphagia and aspiration risk. Third, a single vowel phonation of /e/ was evaluated in this study. Previous studies have shown that the corner vowels /i/, /o/, or /u/ have differences in the acoustic parameters, and the logarithmic energy during vowel phonation was also proven to show differences^41^. The acoustic parameters from this vowel showed good AUC values, but whether multiple vowels can help increase the accuracy is a topic that warrants more future studies. Finally, despite the high AUC, the sensitivity levels for the second algorithm showed wide confidence intervals. Future studies that combine voice signals with other patient-generated health data to further increase these diagnostic properties are warranted.

In conclusion, voice parameters obtained via a mobile device under controlled settings can help to classify those at risk of respiratory complications with high sensitivity and accuracy levels. Whether our novel algorithms using mobile devices can help identify those at a high risk of respiratory complications, allowing for early referral to respiratory experts and subsequently reducing aspiration pneumonia is a topic that needs to be explored in future large-scale multi-center prospective studies.

## Supplementary Information

Supplementary Information.

## Data Availability

Data may be available upon special request to the corresponding authors. Scripts, including the method of feature extract, the preprocessing method, and ML algorithms, on GitHub: https://github.com/ruaeh/Dysphagia-ML.

## Abbreviations

APQ: Amplitude perturbation quotient
AUC: Area under the curve
CI: Confidence interval
CPP: Cepstral peak prominence
DT: Decision tree
F0: Fundamental frequency
FEES: Fiberoptic endoscopic evaluation of swallowing
GMM: Gaussian mixture model
HNR: Harmonic-to-noise ratio
LR: Logistic regression
ML: Machine learning
MBI: Modified Barthel index
MMSE: Mini-mental state examination
NIHSS: National Institutes of Health Stroke Scale
NHR: Noise-to-harmonic ratio
RAP: Relative average perturbation
RF: Random forest
PCF: Peak cough flow
PPQ5: Five point-period perturbation quotient
SNR: Signal-to-noise ratio
SVM: Support vector machine
VFSS: Videofluoroscopic swallowing study
VIF: Variance inflation factor
XGBoost: EXtreme gradient boost
