# Author Correction: Post-stroke respiratory complications using machine learning with voice features from mobile devices

**DOI:** 10.1038/s41598-022-26224-9

**Published:** 2022-12-16

**Authors:** Hae-Yeon Park, DoGyeom Park, Hye Seon Kang, HyunBum Kim, Seungchul Lee, Sun Im

**Affiliations:** 1 Department of Rehabilitation Medicine, Seoul St. Mary’s Hospital, College of Medicine, The Catholic University of Korea, Seoul, Republic of Korea; 2 Graduate School of Artificial Intelligence, Pohang University of Science and Technology (POSTECH), Pohang, Republic of Korea; 3 Department of Pulmonary, Allergy and Critical Care Medicine, Bucheon St. Mary’s Hospital, College of Medicine, The Catholic University of Korea, Seoul, Republic of Korea; 4 Department of Internal Medicine, Bucheon St. Mary’s Hospital, College of Medicine, The Catholic University of Korea, Seoul, Republic of Korea; 5 Department of Otolaryngology-Head and Neck Surgery, Yeouido St. Mary’s Hospital, College of Medicine, The Catholic University of Korea, Seoul, Republic of Korea; 6 Department of Mechanical Engineering, Pohang University of Science and Technology (POSTECH), 223, 5th Engineering Building, 77 Cheongam-Ro, Nam-Gu, Pohang, 37673 Gyeongbuk Republic of Korea; 7 Department of Rehabilitation Medicine, Bucheon St. Mary’s Hospital, College of Medicine, Catholic University of Korea, 327 Sosa-ro, Seoul, Bucheon-si, 14647 Gyeonggi-do Republic of Korea

Correction to: *Scientific Reports* 10.1038/s41598-022-20348-8, published online 06 October 2022

The original version of this Article contained an error in the Author Information section.

“These authors contributed equally: Hae-Yeon Park, DoGyeom Park, Seungchul Lee and Sun Im.”

now reads:

“These authors contributed equally: Hae-Yeon Park and DoGyeom Park.

These authors jointly supervised this work: Seungchul Lee and Sun Im.”

In addition, the Article contained an error in the Funding section.

“This work was supported by a National Research Foundation of Korea (NRF) grant funded by the Korean government (MSIT) (no. 2020R1F1A106581412), the by the Po-Ca Networking Groups funded by The Postech-Catholic Biomedical Engineering Institute (No. 5-2020-B0001-00050) and by Priority Research Centers Program through the National Research Foundation of Korea (NRF) funded by the Ministry of Education (2020R1A6A1A03047902).”

now reads:

“This work was supported by a National Research Foundation of Korea (NRF) grant funded by the Korean government (MSIT) (no. 2020R1F1A106581413), by the Po-Ca Networking Groups funded by The Postech-Catholic Biomedical Engineering Institute (No. 5-2020-B0001-00050) and by the Priority Research Centers Program through the National Research Foundation of Korea (NRF) funded by the Ministry of Education (2020R1A6A1A03047902).”

The original Article and accompanying Supplementary Information file have been corrected.

