# Supplementary material for: Post-stroke respiratory complications using machine learning with voice features from mobile devices
Source: Sci Rep. 2022 Oct 6;12:16682. doi: 10.1038/s41598-022-20348-8 (PMC9537337; doi:10.1038/s41598-022-20348-8)
Supplement: Supplementary file 1 — Supplementary Information. [file 41598_2022_20348_MOESM1_ESM.docx]

**Post-stroke respiratory complications using machine learning** **with voice features from mobile devices**

Hae-Yeon Park^1*^, DoGyeom Park^2,5*^, Hye Seon Kang^3^, HyunBum Kim^4^, Seungchul Lee^2,5^**^†^** & Sun Im^6^**^†^**

***These two authors contributed equally**

**^†^These authors jointly supervised this work**

**Affiliations of the authors**

^1^Department of Rehabilitation Medicine, Seoul St. Mary’s Hospital, College of Medicine, The Catholic University of Korea, Seoul, Republic of Korea

^2^Graduate School of Artificial Intelligence, Pohang University of Science and Technology (POSTECH), Pohang, Republic of Korea

^3^Department of Pulmonary, Allergy and Critical Care Medicine, Department of Internal Medicine, Bucheon St. Mary’s Hospital, College of Medicine, The Catholic University of Korea, Seoul, Republic of Korea

^4^Department of Otolaryngology-Head and Neck Surgery, Yeouido St. Mary’s Hospital, College of Medicine, The Catholic University of Korea, Seoul, Republic of Korea

^5^Department of Mechanical Engineering, Pohang University of Science and Technology (POSTECH), Pohang, Republic of Korea

^6^Department of Rehabilitation Medicine, Bucheon St. Mary’s Hospital, College of Medicine, The Catholic University of Korea, Seoul, Republic of Korea

**^†^Correspondence:** [seunglee@postech.ac.kr](mailto:seunglee@postech.ac.kr) (Seungchul Lee); [lafolia@catholic.ac.kr](mailto:lafolia@catholic.ac.kr) (Sun Im)

**Corresponding author:**

Sun Im, MD, Ph.D.; Department of Rehabilitation Medicine, Bucheon St. Mary’s Hospital, College of Medicine, Catholic University of Korea, 327 Sosa-ro, Bucheon-si, Gyeonggi-do, 14647 Republic of Korea.

E-mail: lafoliamd@gmail.com, lafolia@catholic.ac.kr

Seungchul Lee, Ph.D.; Department of Mechanical Engineering and Graduate School of Artificial Intelligence, Pohang University of Science and Technology (POSTECH), 223, 5th Engineering Building, 77 Cheongam-Ro, Nam-Gu Pohang, Gyeongbuk, 37673 Korea.

E-mail: [seunglee@postech.ac.kr](mailto:seunglee@postech.ac.kr)

**Supplementary Methods S1.**

Instrumental swallowing assessment was performed either with the videofluoroscopic swallowing study (VFSS) or, in patients who could not undergo VFSS, fiberoptic endoscopic evaluation of swallowing (FEES)^1^. The functional oral intake scale^2^ and the penetration-aspiration scale^3^ were recorded. All instrumental swallowing assessments were performed by a certified specialist with over ten years of experience in both the FEES and VFSS. Clinical swallowing assessments were performed using the Mann assessment of swallowing ability^4^. Functional assessment included the National Institutes of Health Stroke Scale (NIHSS) at the time of swallowing assessment^5^, modified Barthel index (MBI) ^6^, mini-mental state examination (MMSE) ^7^, and Berg balance scale^8^.

**Supplementary Methods S2. The preprocessing steps of data splitting, transformation and performance evaluation**

*Data Split*

The dataset used does not exhibit data imbalance for each class, but the amount of data for males and females differs. Gender influences the distribution range of voice parameters. Consequently, the ML algorithms may only perform classification based on gender. We configured a test dataset with the same amount of data for each category to check whether overfitting occurred and split our dataset into training and test sets, where the test set consisted of 20% of the dataset. To measure generalized performance, bootstrap sampling, which uses random sampling with replacement, was repeated ten times. The performance matrix represented the 95% confidence interval (CI) for the average of all results.

*Transformation*

Considering different conditions for each patient, two preprocessing steps were introduced to extract features of the voice signal under the same conditions. First, normalization is implemented to change the value of the voice signal to a common scale. When the voice is recorded, the distance from the microphone may be different for each patient. To alleviate these differences, each signal was divided by the maximum absolute value of the recording per patient while considering peak outliers. Second, we removed the rest time by detecting only phonation. While patients repeated the /e/ phonation three times, there may be a difference in rest time for each patient, which may affect the feature extraction process. The duration of silence was measured using the spectral flux, which is a measure of how quickly the power spectrum of a signal changes. The accuracy, sensitivity, specificity, negative predictive value, positive predictive value, F1 score, and area under the curve (AUC) values were used as performance metrics with emphasis put on high sensitivity levels.

*Feature Preprocessing*

Audio features extrated from voice of patient and clinical variable have different scale. Therefore, Min-Max Scaling is applied for normalization that is defined:

$X=\frac{x-\min\left( x \right)}{\max\left( x \right)-\min\left( x \right)}$ , (1)

where $\max\left( x \right)$ and $\min\left( x \right)$ represent the maximum value of $x$ and minimum value of $x$, respectively. All feature as input of machine learning algorithms are adjusted in the range of 0 to 1. Normalizing technic makes all features set to the same scale, supports faster learning, and prevents over-fitting

*Performance*

Performance was evaluated using a 95% confidence interval for the mean of ten bootstrap samples. To prevent overfitting issues, we used two techniques, regularization methods and feature selection. The regularization technique discourages learning a more complex model, so improving the model’s performance on the unseen data. The feature selection method is intended to reduce the number of input features, resulting in enhanced target variable prediction on training data.

**Supplementary Methods S3. Descriptions of features extracted using Praat software**

The local jitter is the average absolute difference between two consecutive periods, divided by the average interval, and the local absolute jitter uses the absolute difference. The RAP jitter is the relative average perturbation of itself and the two adjacent neighbors, whereas the PPQ5 jitter accounts for four neighbors. Ddp jitter indicates average absolute difference between consecutive differences between consecutive periods, divided by the average period. The local shimmer is the average absolute difference between the amplitudes of two consecutive periods, divided by the average amplitude, and localdbShimmer uses the base of 10 logarithms. APQ is the mean absolute difference between the amplitude of a period and the mean of its amplitude, and that of its closest neighbors divided by the general mean amplitude. The values 3, 5, and 11 of the APQ parameter represent the number of periods used to calculate the average. Dda shimmer is the mean absolute difference between consecutive amplitude differences of consecutive periods. The CPP is an acoustic measure that reflects dysphonia severity. Background noise reflected by the signal-to-noise ratio (SNR) was obtained. The accepted level of SNR should be at least 30 dB for accurate perturbation measurements^9^.

**Feature Selection**

The jitter parameters are defined as the frequency variation from cycle to cycle. Likewise, the shimmer parameters contain similar information as the amplitude variation of the sound wave. These are highly correlated. Thus, collinearity or multicollinearity occurs if all these parameters are simultaneously used for ML. Because collinearity prevents the design of general and robust models in ML, the variance inflation factor was introduced as an indicator with a large value treated as collinearity. We ranked the variables according to variance inflation factor (VIF) value and repeatedly eliminated the variable with the largest value. Ultimately, five features were selected: i) the average value and ii) standard deviation of the fundamental frequency (F0) for fundamental frequency parameters; iii) harmonic-to-noise ratio (HNR) for noise parameter factors; iv) RAP for frequency perturbation parameters, and v) APQ11Shimmer for amplitude perturbation parameters factors, respectively.

**Supplementary Methods S4. Machine Learning Algorithms**

There is a field of machine learning that focuses on studying human voice. Many ML algorithms have been used to detect voice disorder ^10^. We used the following algorithms for ML classification: Logistic Regression (LR), Decision Tree (DT), Random Forest (RF), Support Vector Machine (SVM), Gaussian Mixture Model (GMM), and Extreme Gradient Boost (XGBoost). We implemented LR, DT, RF, SVM, GMM using the *scikit-learn* python library. XGBoost was implemented using the *xgboost* python library. We provide hyperparameters for each ML algorithm in the below ML description. Hyperparameters which is not provided in this section are default setting by *scikit-learn* library.

*Logistic Regression (LR)*^11^: Like a general regression model, the logistic regression model is a statistical model that represents the relationship between the independent and dependent variables as a function. However, there is a difference in that when categorical data are used as an input value, the result is given as the probability of belonging to a specific class. Additionally, unlike the general regression model, which has a dependent variable range of –inf to +inf, the LR model is expressed as an S-shaped logistic function with either 0 or 1 value. As a result, in order to use the LR as a binary classifier, a threshold must be assigned to distinguish between two classes. A probability value greater than 0.5, for example, will classify an input instance as 'class A'; otherwise, 'class B'. The LR model can be extended to model a categorical variable with more than two values which is known as the multinomial logistic regression. In this study, we tuned hyperparameter, ‘lbfgs’ as a solver and L2 regularization as a penalty.

*Decision Tree (DT)*^12^: DT is one of the earliest and prominent machine learning algorithms and a method for classifying or predicting the decision rule using a tree structure. The DT is composed of nodes. Starting at the root node, a child node is generated until each branch reaches the terminal node by the splitting criterion that determines which variable is used and what value of the threshold is used to classify classes. The terminal node is called leaf node and corresponds to the decision outcomes. The process of the model is simpler and more understandable than other ML algorithms. When traversing the tree for classification, the results of each node provide sufficient information to speculate about its class. In this study, we used hyperparameter in which the maximum depth of the tree is expanded until all leaves are pure or until all leaves contain less than two samples and the function to measure the quality of a split is GINI impurity.

*Random Forest (RF)*^12^: Ensemble learning is a model that makes predictions based on several different models. By combining individual models, the ensemble model tends to be more flexible and less data sensitive. RF is an ensemble model that is composed of numerous DTs, similar to how a forest is made up of numerous trees. DTs that have many nodes often cause overfitting of the training data. The various DTs of an RF are trained using the different training dataset components. The input vector of a new sample must pass with each DT of the forest in order to be classified. Then, each DT takes into account a different part of the input vector and provides a classification result. Then, the classification with the most votes or the average of all trees in the forest is then selected. Because the RF considers the outcomes from various DTs, RF can solve the problem caused by taking into account a single DT for the same dataset. In this study, we tuned hyperparmeters in which the number of trees in the forest is 50 and the function to measure the quality of a split is GINI impurity.

*Support Vector Machine (SVM)* ^13^: SVM is able to classify both linear and non-linear data. The first step is to map each data into an *n*-dimensional feature space, where *n* is the number of features. The next step is to find the hyperplane, which is called a decision boundary, that divides data into two classes while minimizing classification errors and maximizing the marginal distance for each class. The data located closest to the hyperplane are support vectors. In this study, we used hyperparmeter, Radial Basis Function (RBF) as a kernel type.

*Gaussian Mixture Model (GMM)*^14^: There are two clustering algorithms, K-means and GMM, widely used in unsupervised ML task. The K-means algorithm for clustering has a number of advantages, including simple implementation, guaranteed convergence, and fast computation time. However, they have a few significant drawbacks. Problems of K-means algorithm is that the data must follow a circular format and data points are assigned to clusters in a deterministic manner, so even if an observation is in an overlapping region, it will be assigned to a single cluster. On the other hand, GMM is a probabilistic clustering technique that can address some of the K-means drawbacks and provides a combination of K Gaussian distributions as a weighted sum of Gaussian density functions. Then, instead of identifying clusters only by their centroids, a set of probability distributions are fitted to the data observations. Consequently, the assumption that the observations are Gaussian distributed is less restrictive than assuming that the clusters are round shaped. In this study, we tuned hyperparameters in which the number of mixture components is 5, the number of Expectation-Maximization (EM) iterations to perform is 100, and parameters are initialized using kmeans algorithm.

*Extreme Gradient Boost (XGBoost)*^15^: XGBoost is a tree-based algorithm that uses the ensemble method. Boosting is a method for converting weak learners into strong learners. Gradient boosting identifies shortcomings by using gradients in the loss function. The major inefficiency in gradient boosting is that it creates one DT at a time. XGBoost is an algorithm that dramatically reduces the training time by enabling gradient boost with parallel learning. It features parallelized tree building, cache-aware access, sparsity awareness, regularization, and weighted quantile sketch as some of its system optimization and algorithmic enhancements. In this study, we tuned hyperparmeters in which learning rate is 0.3 and maximum depth of a tree is 6.

**References**

1. Kidder, T. M., Langmore, S. E. & Martin, B. J. Indications and techniques of endoscopy in evaluation of cervical dysphagia: comparison with radiographic techniques. *Dysphagia* **9**, 256-261 (1994).

2. Crary, M. A., Mann, G. D. & Groher, M. E. Initial psychometric assessment of a functional oral intake scale for dysphagia in stroke patients. *Arch. Phys. Med. Rehabil.* **86**, 1516-1520 (2005).

3. Rosenbek, J. C., Robbins, J. A., Roecker, E. B., Coyle, J. L. & Wood, J. L. A penetration-aspiration scale. *Dysphagia* **11**, 93-98 (1996).

4. Oh, J. C. Reliability and validity of Korean Mann assessment of swallowing ability(K-MASA) [Dissertation]. Seoul: Yonsei University; 2014.

5. Goldstein, L. B. & Samsa, G. P. Reliability of the National Institutes of Health Stroke Scale. Extension to non-neurologists in the context of a clinical trial. *Stroke* **28**, 307-310 (1997).

6. Shah, S., Vanclay, F. & Cooper, B. Improving the sensitivity of the Barthel Index for stroke rehabilitation. *J. Clin. Epidemiol.* **42**, 703-709 (1989).

7. Park, J.-H. & Kwon, Y. C. Modification of the mini-mental state examination for use in the elderly in a non-western society. Part 1. Development of korean version of mini-mental state examination. *Int. J. Geriatr. Psychiatry* **5**, 381-387 (1990).

8. Berg, K. Measuring balance in the elderly: preliminary development of an instrument. *Physiother. Can.* **41**, 304-311 (1989).

9. Švec, J. G. & Granqvist, S. Tutorial and Guidelines on Measurement of Sound Pressure Level in Voice and Speech. *J. Speech. Lang. Hear. Res.* **61**, 441-461 (2018).

10. Hegde, S., Shetty, S., Rai, S. & Dodderi, T. A Survey on machine learning approaches for automatic detection of voice disorders. *J. Voice* **33**, 947.e911-947.e933 (2019).

11. Qawqzeh, Y. K., Bajahzar, A. S., Jemmali, M., Otoom, M. M. & Thaljaoui, A. Classification of Diabetes Using Photoplethysmogram (PPG) Waveform Analysis: Logistic Regression Modeling. *BioMed Res. Int.* **2020**; <http://dx.doi.org/10.1155/2020/3764653> (2020).

12. Che, D., Liu, Q., Rasheed, K. & Tao, X. Decision Tree and Ensemble Learning Algorithms with Their Applications in Bioinformatics in *Software Tools and Algorithms for Biological Systems* (eds. Arabnia, H. R. & Tran, Q.-N.) 191-199 (Springer New York, 2011).

13. Huang, S. *et al.* Applications of Support Vector Machine (SVM) Learning in Cancer Genomics. *Cancer Genomics Proteomics* **15**, 41-51 (2018).

14. Rani, K. U. & Holi, M. S. GMM classifier for identification of neurological disordered voices using MFCC features. *J. Signal Process. Syst.* **4**, 44-51 (2015).

15. Matoušek, J. & Tihelka, D. Using extreme gradient boosting to detect glottal closure instants in speech signal in *ICASSP 2019-2019 IEEE International Conference on Acoustics, Speech and Signal Processing (ICASSP)* 6515-6519 (IEEE, 2019).
